# Altered functional connectivity within the default mode network in two animal models with opposing episodic memories

**DOI:** 10.1371/journal.pone.0202661

**Published:** 2018-09-18

**Authors:** Muhuo Ji, Jiangyan Xia, Xiaohui Tang, Jianjun Yang

**Affiliations:** Department of Anesthesiology, Zhongda Hospital, Medical School, Southeast University, Nanjing, China; Istituto Italiano di Tecnologia, ITALY

## Abstract

Memory enhancement and memory decline are two opposing cognitive performances commonly observed in clinical practice, yet the neural mechanisms underlying these two different phenomena remain poorly understood. Accumulating evidence has demonstrated that the default-mode network (DMN) is implicated in diverse cognitive, social, and affective processes. In the present study, we used the retrosplenial cortex as a seed region to study the functional connectivity within the DMN in two animal models with opposing episodic memories, of which memory enhancement was induced by footshocks to mimic posttraumatic stress disorder (PTSD) and memory decline was induced by lipopolysaccharide (LPS) challenge to mimic sepsis-associated encephalopathy (SAE). Our results showed that LPS challenge and footshocks induced opposing episodic memories. With regard to the imaging data, there were significant differences in the functional connectivity between the retrosplenial cortex and the medial prefrontal cortex (mPFC), insular lobe, left piriform cortex, left sensory cortex, and right visual cortex among the three groups. Post-hoc comparisons showed the LPS group had a significantly increased functional connectivity between the retrosplenial cortex and mPFC as compared with the control group. Compared with the LPS group, the PTSD group displayed significantly decreased functional connectivity between the retrosplenial cortex and the right visual cortex, retrosplenial cortex, insular lobe, left piriform cortex, and left sensory cortex. In summary, our study suggests that there is a significant difference in the functional connectivity within the DMN between SAE and PTSD rats.

## Introduction

Memory enhancement and memory decline are two forms of cognitive dysfunctions commonly observed in clinical practice, but to some extent with opposing cognitive performance [1–8]. Memory enhancement such as fear memory, is one of the core symptoms in posttraumatic stress disorder (PTSD) [2,3], whereas memory decline is more frequently reported, such as Alzheimer's disease (AD), stroke, and other neurodegenerative diseases [5–8]. However, the neural mechanisms underlying the opposing memories remain unclear.

Resting-state functional connectivity based on blood oxygen level–dependent (BOLD) functional magnetic resonance imaging (fMRI) is a useful technique that has been extensively applied to study the brain and its functional organization in both healthy and disease states [9–11]. The resting-state functional connectivity is defined as the temporal correlations between spatially remote neurophysiological processes and has been extensively used to investigate the functional alterations of the brain under different conditions [7]. Among the brain networks, the default-mode network (DMN) which mainly includes the precuneus, posterior cingulate cortex, inferior parietal, medial prefrontal cortex (mPFC), and hippocampus, has been implicated in cognitive, social, and affective impairments associated with many neuropsychiatric disorders [12–15]. However, it remains unclear whether functional connectivity within the DMN differs between these two opposing memory performance, such as fear memory and memory decline.

Recent imaging study demonstrates that the rat brain has a DMN activity similar to that in humans [16], providing a useful preclinical model for both physiological and pathophysiological studies. Indeed, it has been reported that DMN aberrations are observed in neuropsychiatric conditions in both mouse and rat models [17, 18]. To take full advantage of the preclinical models, we established two animal models with opposing memories, of which memory enhancement was induced by footshocks to mimic PTSD and memory decline was induced by lipopolysaccharide (LPS) injection to mimic sepsis-associated encephalopathy (SAE). In the present study, we hypothesized that relative to controls, animals with these two opposing episodic memories show differences in brain resting-state fMRI functional connectivity within the DMN.

## Materials and methods

### Animals and study protocol

**Fig 1:**
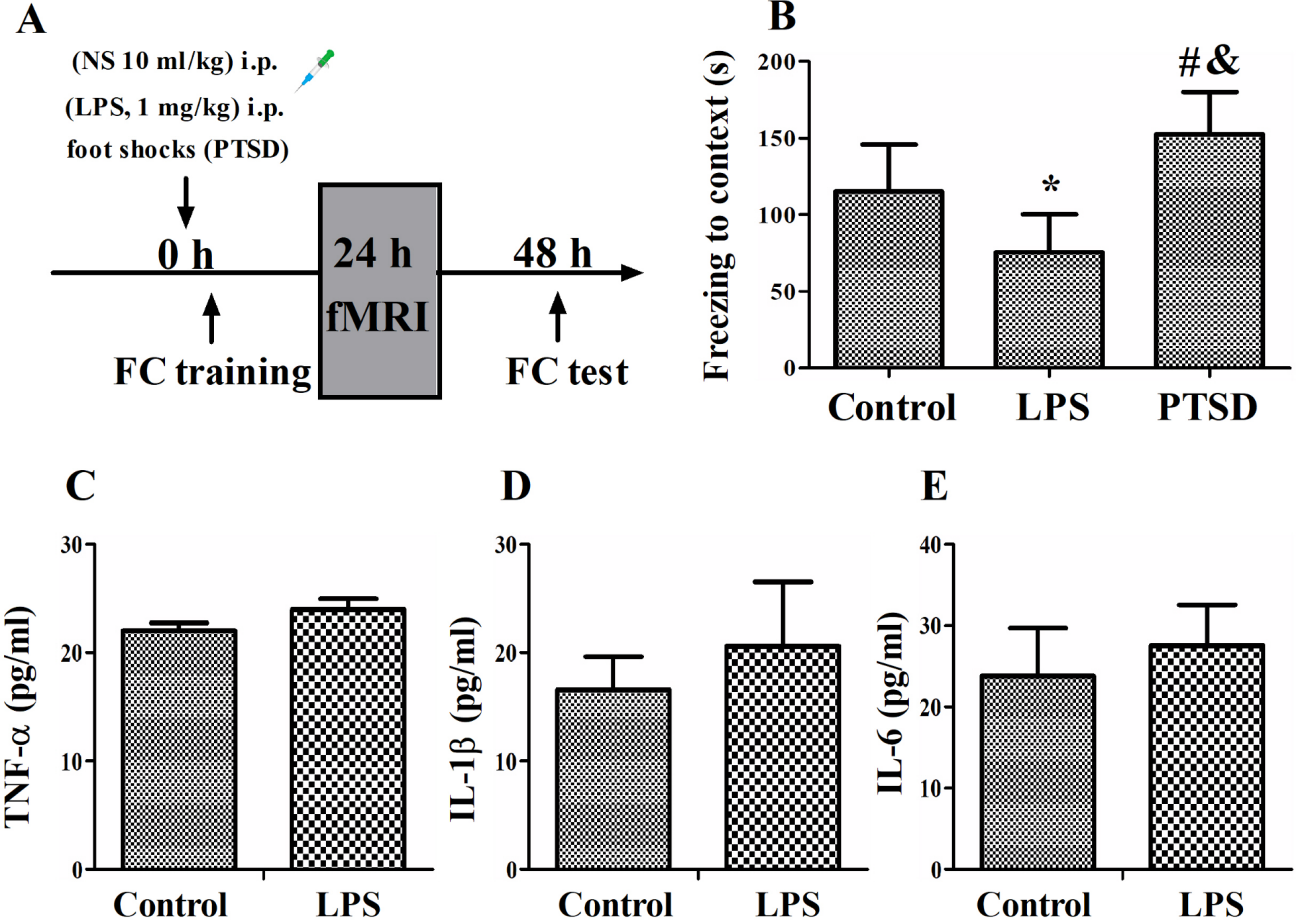
(A) Experimental design of the study. LPS challenge and footshock induced opposing cognitive performance. (B) Rats in the LPS group showed significantly decreased while rats in the PTSD group displayed significantly increased freezing time in the contextual fear conditioning. FC, fear conditioning. NS, normal saline; LPS, lipopolysaccharide. *P < 0.05 vs control group; #*P* < 0.05 vs control group; &*P* < 0.05 vs LPS group. (C-E) LPS challenge did not alter plasma proinflammatory mediator levels, including TNF-α, IL-1β, and IL-6, n = 10 for each group.

All the animal experiments were approved by the Ethics Committee of Zhongda Hospital, Medical School, Southeast University, Nanjing, China, and was conducted in accordance with the Guide for the Care and Use of Laboratory Animals from the National Institutes of Health (Bethesda, MD, USA). Fifty male Sprague-Dawley rats (330–380 g) were purchased from the Animal Center of Jinling Hospital, Nanjing, China, and were reared in conditions with a 12-h light–dark cycle (lights on at 07:00) at 24 ± 1°C. Standard rat chow and water were available *ad libitum* throughout the experiments. In the present study, one rat in the control group was excluded due to obvious head movement, and one rat in the LPS group had brain tumor, thus leaving 15 rats in the control group, 16 rats in the LPS group, and 17 rats in the PTSD group in the final analysis. The flowchart of the experimental protocol was illustrated in Fig 1A.

### SAE animal model

Rats received intraperitoneal injection of either LPS (Escherichia coli endotoxin 0111: B4, Lot # 064M4125V, Sigma, Shanghai, China, 1 mg/kg) or equal volume of normal saline (0.9%). Injections for all animals were given from 8:00 A.M. to 9:00 A.M to exclude the circadian effects. The dose of LPS was selected because it caused neurobehavioral abnormalities but did not result in animal death during our observational period [19, 20].

### PTSD animal model

PTSD model was established as we previously described [3]. Briefly, the rats were acclimated in the chamber for 5 min and then received five electric footshocks (2 mA, 2 s) that were randomly divided over a 15-minute period. Then, the rat was kept in the chamber for another 60 s before it was returned to home cage. For the controls, animals were left undisturbed in their home cage. After each session, the chamber was cleaned with 75% ethanol.

### Resting-state fMRI acquisition

All animals were scanned by the 7.0 T Bruker Pharmascan MRI scanner (70/16 PharmaScan, Bruker Biospin GmbH, Germany) by a 38 mm birdcage rat brain quadrature resonator for radiofrequency transmission and reception. Anesthesia was induced with 5% isoflurane in oxygen and air (1:1) in a chamber. The animals were then fixed in prone position on the MRI bed and anesthesia was maintained by isoflurane in oxygen via a nose mask with a bite bar. During the MRI scan, the rat was prostrated on a custom-made holder to minimize head motion. During anesthesia maintenance, we used an anesthesia regime consisting of an initial subcutaneous injection of dexmedetomidine (0.03 mg/kg), followed by isoflurane inhalation. The anesthesia level was maintained by adjusting isoflurane concentration (0.2~0.5%) to keep the respiration rate to the range between 60 and 80 breaths/min throughout the whole scanning period. An echo-planar imaging (EPI) sequence with the following parameters was used. Matrix size = 64 × 64, flip angle = 30^o^, resolution = 0.5 mm × 0.5 mm, slice thickness = 1.0 mm, slice gap = 0, repetition time (TR) = 2 s, echo time (TE) = 18 ms, volume = 180. Coplanar T2-weighted scan was also acquired in addition to the functional data. Finally, all the images were band-pass filtered in 0.01–0.1 Hz.

The preprocessing steps begin by eliminating the first 10 time points of the data. Then, slice timing and realignment was performed, 0.1 mm and 1 degree in max head motion was set as excluding criteria. Rat images were registered to a template set based on a standard rat brain atlas of Paxinos and Watson [21]. After spatial normalization, the voxel of fMRI images was resampled to 3 mm × 3 mm ×3 mm. Then spatial smoothness with an isotropic Gaussian kernel (FWHM = 4 mm) was performed on the spatial transformed rat images. A band-pass filter with a cutoff at 0.1 and 0.01 Hz was applied to the time courses of all voxels.

The functional connectivity analysis was processed with Resting State fMRI Data Analysis Toolkit V1.8 software (REST, http://www.restfmri.net). Functional connectivity maps within the DMN were constructed for each animal by placing a seed in the retrosplenial cortex. Pearson correlation was used to compute the functional connectivity value voxel by voxel. In the first place, one sample *t*-test was performed to calculate the T maps of DMN in each group (FDR correction was used, *q* = 0.01). We used FDR rather than family wise error rate correction (FWER) for multiple comparison correction in the one-sample T test in order to detect possible related brain regions within the DMN. Secondly, a mask was constructed by union of DMN masks of three groups. Multiple comparison correction was performed by using FWER correction with a cluster defining threshold *P* = 0.001 (two-tailed), corresponding to a cluster level of *P* = 0.05.

### Fear conditioning test

Fear conditioning test was conducted using a dedicated conditioning chamber as previously described with some modifications [3,7]. It is a widely accepted paradigm to measure contextual memory [2,3,7,8]. A well-trained investigator who was blinded to the animal grouping performed the behavioral tests. The behavior of rats was recorded using a video camera and the apparatus used in tests were purchased from the Shanghai Softmaze Information Technology Co., Ltd., China. In the training section, rats were placed into the conditioning chamber (32 cm × 25 cm × 25 cm) 30 min after LPS injection. The rats were allowed to explore the chamber for 3 min before the onset of a 30-s tone (70 db, 3 kHz), followed by a 2-s footshock (0.75 mA). Then, the animals remained in the chamber for another 30 s and were then returned to their home cages. Forty-eight hours after training, the contextual memory (a hippocampus-dependent task) was tested by returning the rats to the same chamber in which training occurred, and freezing behavior was recorded for 5 min without tone presentation or footshock during this period. Freezing behavior was defined as the absence of all visible movement, excluding respiration.

### Enzyme-linked immunosorbent assay (ELISA)

Following deep anesthesia with 2% sodium pentobarbital in saline (60 mg/kg, intraperitoneally; Sigma Chemical Co., St. Louis, MO, USA), blood was taken transcardially after thoracotomy and centrifuged at 5000 rpm for 5 min at 4°C. Plasma samples were stored at -80°C for further analysis. The levels of tumor necrosis factor (TNF-α), interleukin-1β (IL-1β), and IL-6 were measured by commercially available ELISA kits from JianCheng Biotechnology, Nanjing, China.

### Statistical analysis

Data analysis was performed using SPSS for Windows software (Version 19.0; SPSS, Chicago, IL). Data are shown as mean ± standard deviation (S.D.). Data for behavioral test and proinflammatory mediators were tested for normal distribution by the Shapiro-Wilk test. Comparisons between two groups were performed by independent-samples t-tests. One-way analysis of variance (ANOVA) and then Bonferroni corrections were used for multiple comparisons. One-sample t tests were performed on functional connectivity maps within the DMN. To determine the relationship between functional connectivity within DMN and contextual freezing time, Pearson’s correlations were performed. A *P* < 0.05 was considered statistically significant.

## Results

### LPS challenge and footshocks differently regulated memory

Compared with the control group, rats in the LPS group showed significantly decreased freezing time, whereas rats in the PTSD group displayed significantly increased freezing time in the contextual fear conditioning test (Shapiro-Wilk test: *P* = 0.775 for control group, *P* = 0.548 for LPS group, and *P* = 0.052 for PTSD group; F_(2, 45)_ = 32.108, *P* < 0.001, Fig 1B). This suggests we have established two animal models with opposing episodic memories.

### LPS challenge did not alter plasma proinflammatory mediators

LPS challenge did not change plasma levels of proinflammatory mediators compared with the control group (Shapiro-Wilk test: *P* = 0.063 for control group and 0.803 for LPS group; TNF-α: t = -1.623, *P* = 0.122, Fig 1C; Shapiro-Wilk test: *P* = 0.349 for control group and 0.245 for LPS group; IL-1β: t = -1.895, *P* = 0.074, Fig 1D; Shapiro-Wilk test: *P* = 0.974 for control group and 0.499 for LPS group;; IL-6: t = -1.556, *P* = 0.137, Fig 1E).

### Group differences in functional connectivity within the DMN

**Fig 2:**
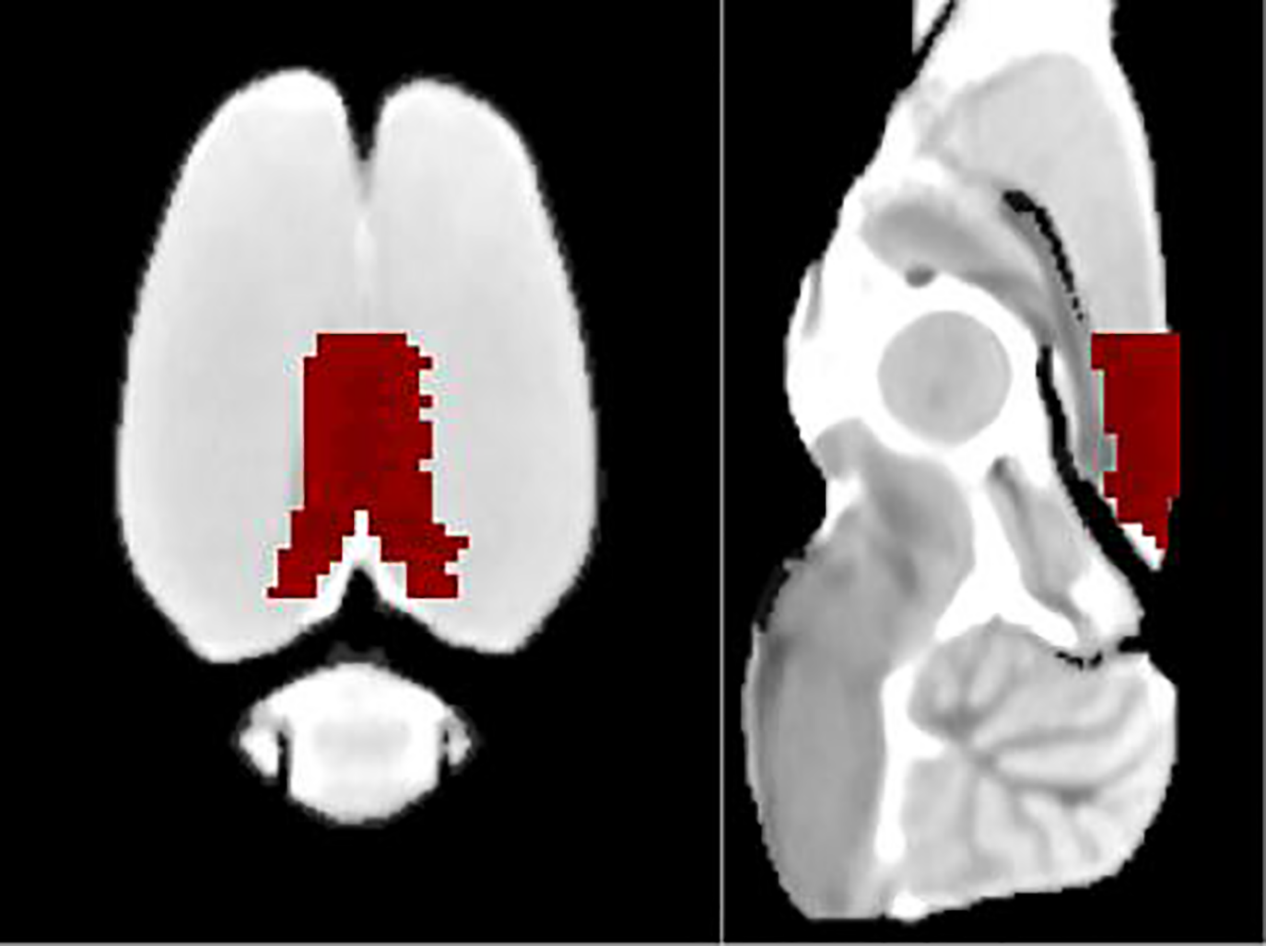
Illustration of the use of seed voxels. An example of the retrosplenial cortex template projected on a coronal (left) or sagittal MR image (right).

**Fig 3:**
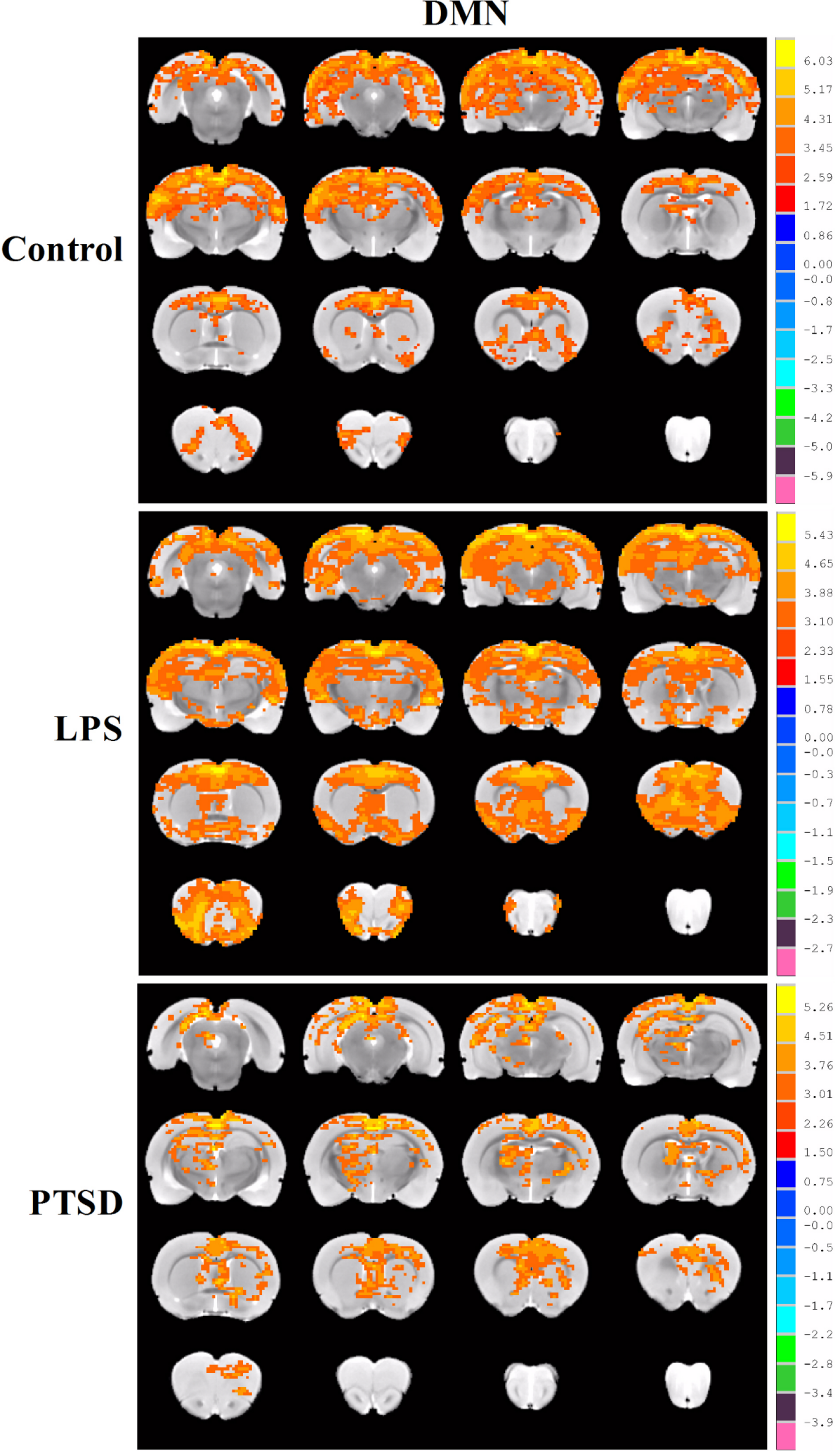
The DMN maps derived from retrosplenial cortex seed. Results from the one-sample t tests for the functional connectivity within the DMN in control, LPS, and PTSD groups. As compared with the control group, the functional connectivity within the DMN was higher in the LPS group, but there was a trend toward a decreased functional connectivity within the DMN in the PTSD group.

We used the retrosplenial cortex as a seed region (Fig 2) to study the functional connectivity within the DMN. The one-sample t test showed the functional connectivity within the DMN map derived from retrosplenial cortex seed of the control, LPS, and PTSD groups. As compared with the control group, the functional connectivity within the DMN was higher in the LPS group, but there was a trend toward a decreased functional connectivity within the DMN in the PTSD group (Fig 3).

**Fig 4:**
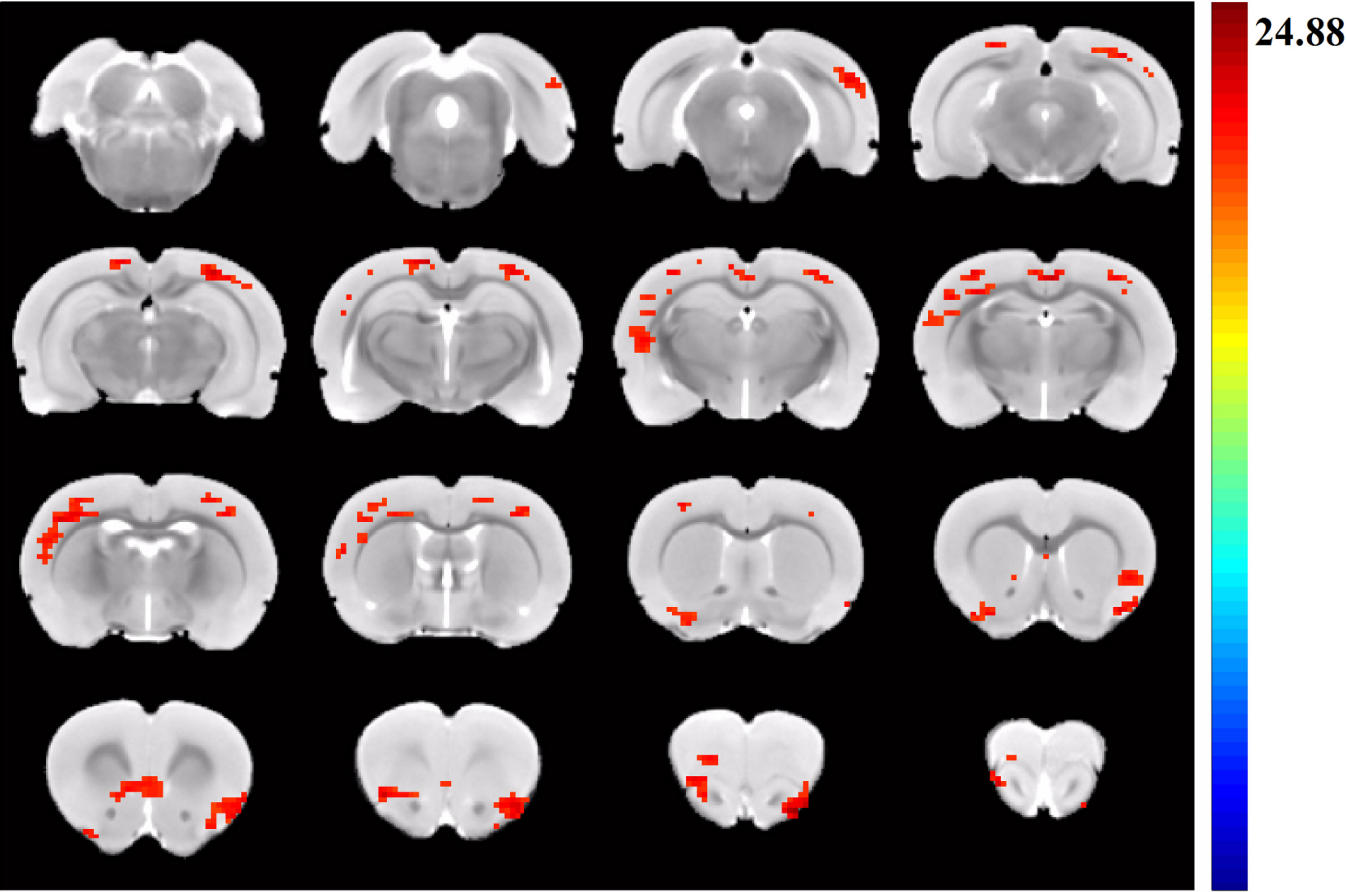
Group differences resulting from the comparison of DMN components in control, LPS, and PTSD groups. There were significant differences in the functional connectivity between retrosplenial cortex and the right visual cortex, retrosplenial cortex, left sensory cortex, left piriform cortex, and insular lobe among the control, LPS, and PTSD groups.

As shown in Fig 4, there were significant differences in functional activities among the control, LPS, and PTSD groups, including the right visual cortex, retrosplenial cortex, left sensory cortex, left piriform cortex, and insular lobe. Table 1 showed Montreal Neurological Institute (MNI) coordinates of peak significance from higher functional connectivity within the DMN among the control, LPS, and PTSD groups.

**Table 1:** Clusters of one-way ANOVA analysis of default-mode network among the three groups (Threshold z = 3.29).

Structural name	Volume (voxels)	z-peak	Peak coordinate		
			x	y	z
Retrosplenial cortex					
Right v2	51	4.0027	-5.7	3.6	-6.6
Right v1	217	4.3922	-3.3	5.4	-4.8
Retrosplenial cortex	124	4.8947	1.5	6.0	-4.2
Left sensory cortex	414	4.3567	4.5	5.4	-2.4
Left piriform cortex	85	3.9179	3.3	-1.5	1.5
Right insular	235	5.3164	-2.1	0	4.5
Left insular	215	4.6238	3.3	1.8	4.5

**Fig 5:**
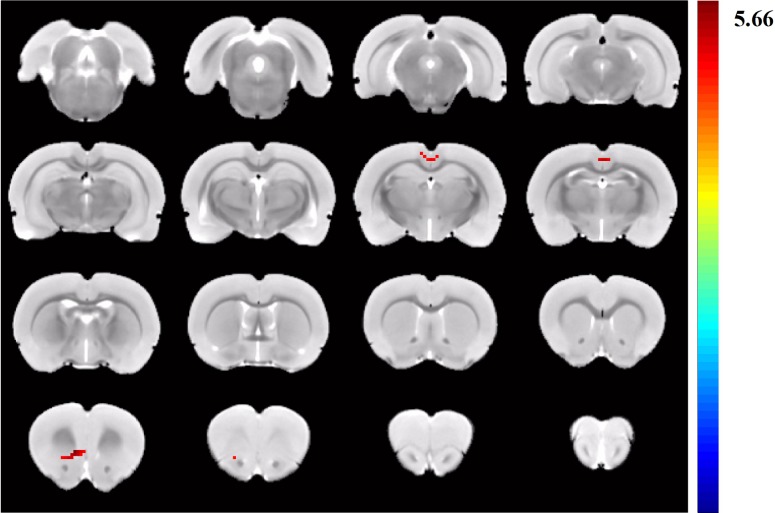
Group differences resulting from the comparison of DMN components between LPS group and control group. The LPS group showed a significantly increased functional connectivity between the retrosplenial cortex and mPFC when compared with the control group.

Post-hoc comparisons of the three groups were further calculated. The LPS group showed a significantly increased functional connectivity between the retrosplenial cortex and medial prefrontal cortex (mPFC) compared with the control group (Fig 5). Table 2 displayed MNI coordinates of peak significance from higher functional connectivity within the DMN between the control and groups.

**Table 2:** Clusters of post-hoc analysis within the default-mode network between the control and LPS groups (Threshold z = 3.31).

Structural name	Volume (voxels)	z-peak	Peak coordinate		
			x	y	z
Retrosplenial cortex					
Retrosplenial cortex	29	4.1525	0.9	5.4	-2.1
Medial prefrontal cortex	43	4.6092	1.8	1.5	2.4

**Fig 6:**
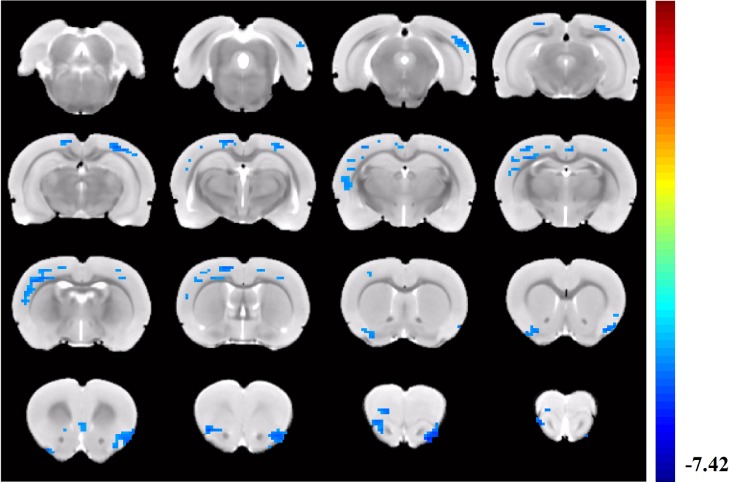
Group differences resulting from the comparison of DMN components between LPS group and PTSD group. Compared with the LPS group, the retrosplenial cortex showed significantly decreased functional connectivity to the left visual cortex, retrosplenial cortex, insular lobe, and left piriform cortex.

Compared with LPS group, PTSD group showed decreased functional connectivity in the left visual cortex, retrosplenial cortex, insular lobe, and left piriform cortex (Fig 6). Table 3 revealed MNI coordinates of peak significance from lower functional connectivity within the DMN between the LPS and PTSD groups.

**Table 3:** Clusters of post-hoc analysis within the default-mode network between the LPS and PTSD groups (Threshold z = -3.31).

Structural name	Volume (voxels)	z-peak	Peak coordinate		
			x	y	z
Retrosplenial cortex					
Left v2	113	-4.2538	-3.3	5.4	-4.8
Retrosplenial cortex	95	-4.616	1.5	6.0	-4.2
Left s1	334	-4.1921	4.2	4.2	-1.8
Left piriform cortex	66	-4.3646	3.9	-1.2	2.4
Left insular	213	-5.5849	-2.1	0	4.5
Right insular	125	-4.7013	3.0	1.5	4.8

### Correlational analyses

**Fig 7:**
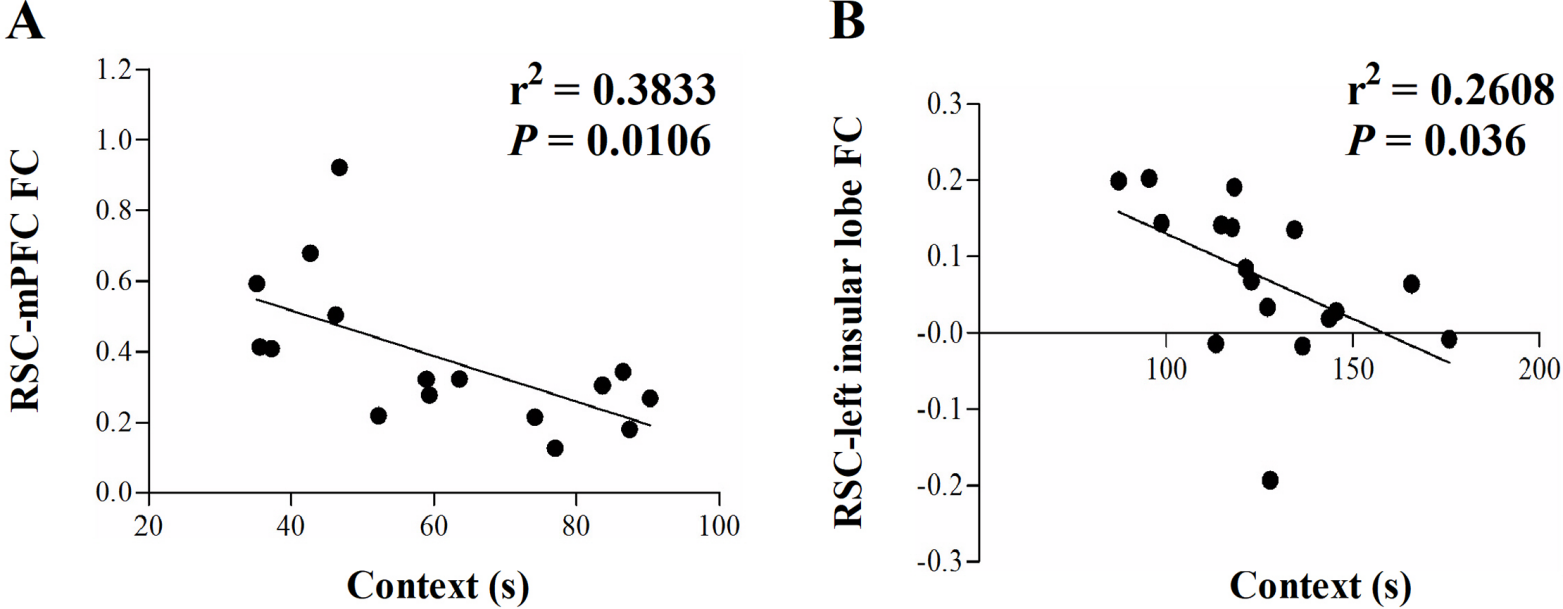
Relationship between contextual freezing time and functional connectivity. Within the LPS group, an association between the contextual freezing time and functional connectivity between the retrosplenial cortex and mPFC was observed (a). Within the PTSD group, there was a significant association between the contextual freezing time and functional connectivity between the retrosplenial cortex and insular lobe (b).

We extracted the mean functional connectivity values within clusters showing significant difference among three groups and correlated them with contextual freezing time. Multiple comparisons were corrected using FDR (*q* = 0.05). Within the LPS group, there was a negative correlation between the contextual freezing time and functional connectivity between the retrosplenial cortex and mPFC (Fig 7A). Within the PTSD group, a negative correlation was observed between the contextual freezing time and functional connectivity between the retrosplenial cortex and left insular lobe (Fig 7B). However, there was no correlation between the contextual freezing time and functional connectivity within the DMN in the control group (data not shown).

## Discussion

In the present study, we used resting-state fMRI to test the hypothesis that the opposing episodic memories induced by LPS challenge and footshocks differ in the functional connectivity within the DMN. We found that LPS-exposed rats are characterized by an increase in functional connectivity between retrosplenial cortex and mPFC, whereas PTSD rats displayed decreased functional connectivity between the retrosplenial cortex and the right visual cortex, retrosplenial cortex, insular lobe, left piriform cortex, and left sensory cortex.

PTSD is a psychiatric disorder that may occur after intensely psychological trauma or physiological stress [1–3]. Although many animal models of PTSD have been proposed [22], foot-shock is one of the most common aversive stressors used in rodent fear models, mainly to assess acute stress responses and fear learning [2]. On the other hand, memory decline is more frequently observed in clinical situations, including AD, stroke, and other neurodegenerative diseases [5–8]. Since SAE and PTSD share an overlap of many symptoms, it is not an easy task to develop two animal models with perfect opposing cognitive performance. However, there is accumulating evidence suggesting SAE and PTSD shows opposing episodic memories [2,3,8]. To take full advantage of preclinical models, we adopted two animal models with opposing memories. One is PTSD with enhanced memory, which is induced by footshocks. The other one is SAE with memory decline, which is induced by LPS challenge. We selected these two animal models because they can induce opposing episodic memories and are also easy to implement. Consistently, we showed that LPS challenge and footshocks induced opposing episodic memories, with decreased and increased freezing time in the contextual fear conditioning in the LPS group and PTSD group, respectively. However, the neural mechanisms underlying these two opposing memories remain unclear.

Resting-state fMRI has been extensively applied to study the neural activity of human brain [7]. In resting-state fMRI, the DMN is believed to reflect spontaneous neural activity, which shows a high level of activity during rest and deactivates during external cognitive tasks [23–25]. The rat DMN includes cingulate cortex, prelimbic cortex, orbital cortex, retrosplenial cortex, posterior parietal cortex, secondary visual cortex, hippocampus, and auditory cortex, which plays a key role in spontaneous cognition [16]. Recent studies also implicate the DMN in core working memory processes [26, 27], offering an explanation for the observed engagement of this network in a wide variety of cognitive tasks. By contrast, dysregulated DMN is associated with various neurological and psychiatric disorders, including AD [28], schizophrenia [13], stroke [14], and depression [23]. In addition, it is reported that DMN regions are involved in a wide spectrum of language processing, visual and auditory attention, and motoric activities [29]. In animal studies, it has been shown that the connectivity within the rat DMN is increased after maze learning but decreased one week later, suggesting that the rodent DMN may be involved in early memory consolidation [30]. Other groups further identified sub-network modules in the rat DMN that display age- and behavior-related decline [31]. Although the cingulate cortex is thought to be a functional hub region conserved from rodents to humans [32–34], there are also studies using retrosplenial cortex, a brain region can be related to human precuneus, as seed for functional connectivity analysis within the DMN [16,31,35]. The strength of interactions within the DMN, particularly between medial temporal lobe and medial-parietal subsystems such as the posterior cingulate cortex and retrosplenial cortex, relates to episodic memory processes [36]. Importantly, retrosplenial cortex is thought to be an important gateway to episodic memory by linking subcortical and cortical subsystems of the DMN in older adults [37]. These findings imply that retrosplenial cortex has a central role in controlling information transfer within the DMN and forms a critical gateway within the DMN to support episodic memory.

To evaluate the DMN of rat, we selected retrosplenial cortex as seed and showed increased functional connectivity within the DMN in the LPS group, but a trend toward a decreased functional connectivity in the PTSD group. This suggests that the DMN in the LPS group has higher inter-coupling than that in the PTSD group. Although there is a paucity of information available on the functional connectivity changes within DMN after peripheral LPS challenge, previous neuroimaging studies have demonstrated that systemic inflammation alters the functional connectivity of brain networks within the human brain at rest, most of them belong to DMN [38–40]. Our finding that increased functional connectivity between retrosplenial cortex and mPFC in the LPS group, potentially indicative of a neural compensatory mechanism to offset functional impairments. It has been suggested that brain region such as mPFC is implicated specifically in regulating autonomic and neuroendocrine processes that relate to systemic inflammation via bidirectional signaling mechanisms [38], providing an important pathway that affects learning, memory, and emotional behavior.

On the contrary, our study showed decreased functional connectivity within the DMN for PTSD group, compared with LPS group, mainly in the right visual cortex, retrosplenial cortex, insular lobe, left piriform cortex, and left sensory cortex. Indeed, one recent study has suggested decreased functional connectivity within the DMN in PTSD patients [41]. In addition, the severity of PTSD symptoms is associated with reduced DMN connectivity in individuals with elevated genetic risk for psychopathology [42]. Of note, it has been suggested that therapies targeting the aberrant DMN through mindfulness training might help prevent overgeneralization of fear associations [43]. Our correlational analyses in the PTSD group further showed a negative correlation between reduced insular lobe activity and fear memory represented in contextual freezing time. The disrupted intrinsic connectivity of the insular network is associated with abnormal episodic memory in patients with amnesic mild cognitive impairment [44]. A somewhat surprising finding was that although rats in LPS and PTSD groups showed opposing functional connectivity within the DMN, they do not affect the same brain regions using the retrosplenial cortex seed. This discrepant finding might be explained by that SAE and PTSD involve different brain regions with different cognitive domains being affected. Collectively, our study suggested there is a significant difference in the functional connectivity within the DMN in two animal models with opposing episodic memories, but our speculation needs to be confirmed in human studies.

Several limitations should be acknowledged in our study. Firstly, the relatively small sample size may reduce the generalizability of our results to some extent. Secondly, our study was confined to the connectivity between the retrosplenial cortex and other regions within the DMN, while the changes in directed functional connectivity between the retrosplenial cortex and brain regions outside the DMN as well as their impacts have not been explored. In addition, it should be noted that the changes of functional connectivity within the DMN reported from our studies are based on retrosplenial cortex, future experiments using the anterior cingulate as a seed should be considered. Thirdly, it is difficult to perform the resting-state fMRI experiments on conscious animals due to motion artifacts, the strong influence of anesthesia on our results cannot be excluded. Thus, monitoring other physiological parameters such as temperature, heart rate, and cerebral blood flow is necessary in our future studies.

### Conclusion

To the best of our knowledge, the current study was the first to show that differences in functional connectivity within the DMN between SAE and PTSD rats. Our findings have important implications for the understanding of the neural mechanisms underlying different neuropsychiatric disorders. In light of the common resting state brain networks in humans and rats, resting-state fMRI can be used as a translational tool to advance the pathophysiological understanding of disorders related to cognitive dysfunctions.
